# IP-10/CXCL10 induction in human pancreatic cancer stroma influences lymphocytes recruitment and correlates with poor survival

**DOI:** 10.18632/oncotarget.2519

**Published:** 2014-11-25

**Authors:** Serena Lunardi, Nigel B. Jamieson, Su Yin Lim, Kristin L. Griffiths, Manuela Carvalho-Gaspar, Osama Al-Assar, Sabira Yameen, Ross C. Carter, Colin J. McKay, Gabriele Spoletini, Stefano D'Ugo, Michael A. Silva, Owen J. Sansom, Klaus-Peter Janssen, Ruth J. Muschel, Thomas B. Brunner

**Affiliations:** ^1^ Gray Institute for Radiation Oncology and Biology, Department of Oncology, University of Oxford, Oxford, United Kingdom; ^2^ West of Scotland Pancreatic Unit, Glasgow Royal Infirmary, Glasgow, United Kingdom; ^3^ Jenner Institute, University of Oxford, Old Road Campus, Oxford, United Kingdom; ^4^ Hepatobiliary and Pancreatic Surgery, Churchill Hospital, Oxford, United Kingdom; ^5^ Beatson Institute of Cancer Research, Garscube Estate, Glasgow, United Kingdom; ^6^ Department of Surgery, Technische Universitaet Muenchen, 81675 Muenchen, Germany; ^7^ Department of Radiation Oncology, University Hospitals Freiburg, 79106 Freiburg, Germany

**Keywords:** pancreatic cancer, pancreatic stellate cells, tumor stroma, IP-10, CXCL10

## Abstract

Pancreatic ductal adenocarcinoma (PDAC) is characterized by an abundant desmoplastic reaction driven by pancreatic stellate cells (PSCs) that contributes to tumor progression. Here we sought to characterize the interactions between pancreatic cancer cells (PCCs) and PSCs that affect the inflammatory and immune response in pancreatic tumors. Conditioned media from mono- and cocultures of PSCs and PCCs were assayed for expression of cytokines and growth factors. IP-10/CXCL10 was the most highly induced chemokine in coculture of PSCs and PCCs. Its expression was induced in the PSCs by PCCs. IP-10 was elevated in human PDAC specimens, and positively correlated with high stroma content. Furthermore, gene expression of IP-10 and its receptor CXCR3 were significantly associated with the intratumoral presence of regulatory T cells (Tregs). In an independent cohort of 48 patients with resectable pancreatic ductal adenocarcinoma, high IP-10 expression levels correlated with decreased median overall survival. Finally, IP-10 stimulated the *ex vivo* recruitment of CXCR3^+^ effector T cells as well as CXCR3^+^ Tregs derived from patients with PDAC. Our findings suggest that, in pancreatic cancer, CXCR3^+^ Tregs can be recruited by IP-10 expressed by PSCs in the tumor stroma, leading to immunosuppressive and tumor-promoting effects.

## INTRODUCTION

Pancreatic ductal adenocarcinoma (PDAC) is the 10^th^ most common cancer worldwide and ranks fourth in cancer-related mortality in the USA and fifth in the UK [1, 2]. The high incidence of mortality is a consequence of late presentation, frequent inoperability of the cancer and poor response to the currently available pharmacological modalities for PDAC. Nearly all patients diagnosed with PDAC die from the disease, with a 5-year survival rate of less than 5% [1]. The dismal outcome of current therapies underlines the need to better understand pancreatic cancer biology in order to identify novel approaches.

The histology of pancreatic cancer is notable for a prominent desmoplastic reaction predominantly driven by pancreatic stellate cells (PSCs). PSCs, called stellate cells because of their star-shape, can be activated by inflammatory stimuli, injury and cancer. When activated, PSCs lose their vitamin A storage droplets, express vimentin and α-smooth muscle actin and release high levels of extracellular matrix (ECM) proteins and growth factors that promote proliferation, survival and migration of PCCs [3]. Furthermore, this desmoplastic reaction acts as a physical obstacle to drug delivery and impairs the response to radiotherapy [4, 5]. PSCs were also shown to contribute to the immune suppressive environment of pancreatic adenocarcinoma [6]. In particular, it was reported that high levels of Galectin-1, secreted by the PSCs, induced apoptosis of CD4^+^ and CD8^+^ T cells [7]. Another recent study showed that PSCs secreted cytokines such as IL-6 that enhanced the differentiation of peripheral blood mononuclear cells (PBMCs) to myeloid-derived suppressor cells (MDSCs) and promoted their immunosuppressive activity [8]. Furthermore, activated PSCs were suggested to act as a physical barrier by blocking access of CD8+ T cells to cancer cells [9]. However, the mechanisms by which PSCs influence the immunoresponse are not completely understood and remain subject of active research [10, 11].

Because of the prominent desmoplastic reaction in pancreatic cancer, we designed a study to investigate biologically potent secreted chemokines specifically induced by coculture of hPSC with PCC. After comparing a panel of 50 factors produced by PSC alone or in coculture with PCC, IP-10 emerged as a cytokine induced by coculture that could have a significant effect on the immunological microenvironment of the tumors. Consistent with this hypothesis, IP-10 levels correlated with infiltration of regulatory T cells (Tregs) and survival in human PDAC.

## RESULTS

### Proinflammatory cytokines are released by pscs

We characterized the expression of 50 cytokines, growth factors and chemokines in the conditioned media from PSCs and normal fibroblasts (MRC5) using a Multiplex Luminex suspension assay. Human PSC (hPSC) produced substantially higher levels of proinflammatory cytokines and chemokines than MRC5 (Table 1). Nine factors were expressed at least 4-fold higher in hPSC monoculture than in MRC5 monoculture (average concentrations displayed in Suppl. Table 1). These included interleukins: IL-6, IL-8, IL-1Rα, IL-15 and leukemia inhibitory factor (LIF); chemokines: melanoma growth stimulating activity (GRO)-α, Eotaxin, stromal cell-derived factor (SDF)-1α and interferon-γ inducible protein 10 (IP-10/CXCL10). Among these differentially expressed factors, only IL-6, IL-8 and SDF-1α were previously reported to be expressed by PSCs at the protein level [12, 13].

**Table 1:** Factors secreted by PSCs and normal MRC5 fibroblasts Supernatants were collected from hPSC and MRC5 monocultures and the levels of cytokines were measured using Multiplex suspension array. The relative expression of each factor was calculated by relating the average concentration in hPSC supernatants to that of MRC5 supernatant after culturing for 24 hr (*n* = 4). Factors highlighted in bold were significantly increased in supernatants from hPSC compared to those from MRC5. p, p-value; NA, ratio was not applicable (see Suppl. Table 1). t-test followed by Bonferroni's post-test: *, *p* < 0.05; **, *p* < 0.001; ns, not significant.

Analyte	hPSC vs. MRC5 relative expression	p	Analyte	hPSC vs. MRC5 relative expression	p	Analyte	hPSC vs. MRC5 relative expression	p
**Interleukins**			**Chemokines/monokines**			**Growth factors**		
**IL-8**	**60.58**	**	**GROα**	**103.43**	**	M-CSF	14.65	ns
**IL-6**	**> 31.31**	*	**Eotaxin**	**46.22**	**	G-CSF	7.19	ns
**LIF**	**8.28**	**	**SDF-1α**	**37.92**	**	PDGF-BB	6.73	ns
**IL-1Rα**	**6.25**	**	**IP-10**	**8.70**	*	GM-CSF	5.37	ns
**IL-15**	**4.87**	**	MCP-3	5.99	ns	HGF	2.19	
IL-2	4.68	ns	RANTES	3.40		FGF basic	2.16	
IL-17	4.17	ns	MIP-1α	2.31		b-NGF	1.83	
IL-4	3.88		MIF	1.74		SCF	1.51	
IL-2Rα	2.91		MIG	1.60		VEGF	1.39	
IL-16	2.22		CTACK	1.53		SCGF-b	1.03	
IL-9	2.20		MCP-1	NA		**Apoptosis Mediator**		
IL-12(p40)	2.18		MIP-1β	NA		TRAIL	2.71	
IL-1a	2.14		**Inflammatory cytokines**			**Adhesion molecules**		
IL-3	1.96		IFN-γ	6.91	ns	ICAM-1	3.42	
IL-7	1.83		TNF-α	4.63	ns	VCAM-1	1.92	
IL-18	1.71		TNF-β	1.71				
IL-12(p70)	1.31		IFN-α2	1.61				
IL-10	1.29							
IL-13	1.11							
IL-1β	NA							
IL-5	NA							

### IP-10 is induced in coculture of PSCs with PCCs

**Figure 1:**
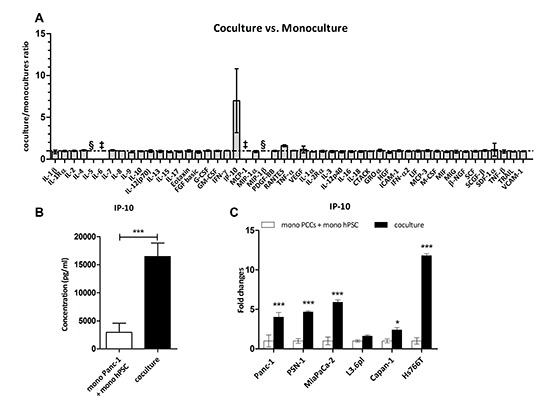
IP-10 is upregulated when PSCs are cocultured with cancer cells **(A)** Expression levels of factors in monocultures and cocultures of Panc-1 and hPSC. Data were quantified using the Multiplex suspension array and expressed as a ratio of coculture concentration divided by the sum from the monocultures for each factor (*n* = 4). §, not detected. ‡, above range of detection. **(B)** IP-10 concentration (pg/mL) in the culture media of the conditions indicated measured in A. t-test: ***, *p* < 0.0001. **(C)** IP-10 levels in supernatants collected after 48 hr. Expression of IP-10 in the coculture was normalized to the sum of monocultures for each of the indicated cell lines (*n* = 4). Two-way ANOVA followed by Bonferroni's post test: *, *p* < 0.05; ***, *p* < 0.0001.

To ask whether the presence of tumor cells changed the expression of cytokines and growth factors secreted by PSCs and vice versa, we examined differences in levels of the secreted factors between the conditioned media of PCCs and PSCs cocultures and monocultures using the same Luminex-based assay. Compared to the monocultures, coculture of Panc-1 cells with hPSC showed a 5-fold increase in IP-10 concentration (Figure 1A-B). Induction of IP-10 was confirmed by ELISA and was observed in cocultures with differing ratios of Panc-1/hPSC (Suppl. Figure 1). The level of IP-10 did not change significantly with increasing proportions of cancer cells.

In addition we examined expression of the same 50 factors from the conditioned media of PSCs cocultured with different pancreatic cancer cell lines (Suppl. Figure 2A-B). Coculture of PSCs with 5 of the 7 cell lines led to increased levels of IP-10. PSN-1 cocultured with hPSC led to a 56-fold increase in IP-10 levels compared to monocultures, coculture with Panc-1, MiaPaCa-2 and Hs766T showed around 4-fold induction, and coculture with Capan-1 showed nearly 2-fold induction (Suppl. Figure 2B). However, no difference in IP-10 expression was observed after coculture with Capan-2 or L3.6pl. We also noted that SCGF-β and HGF were decreased in the cocultures compared to the combined hPSC and PCCs monocultures (Suppl. Figure 2A, C-D). IP-10 expression was confirmed by ELISA with approximately 5-fold increase in IP-10 levels in coculture of hPSC with Panc-1, PSN-1, and MiaPaCa-2, 2-fold with Capan-1, and 12-fold with Hs766T cells compared to the respective monocultures (Figure 1C). Overall, these results suggest that release of IP-10 is a common but not universal consequence of PCCs and PSCs cocultures.

### PCCs secrete soluble factors promoting ip-10 expression by pscs

**Figure 2:**
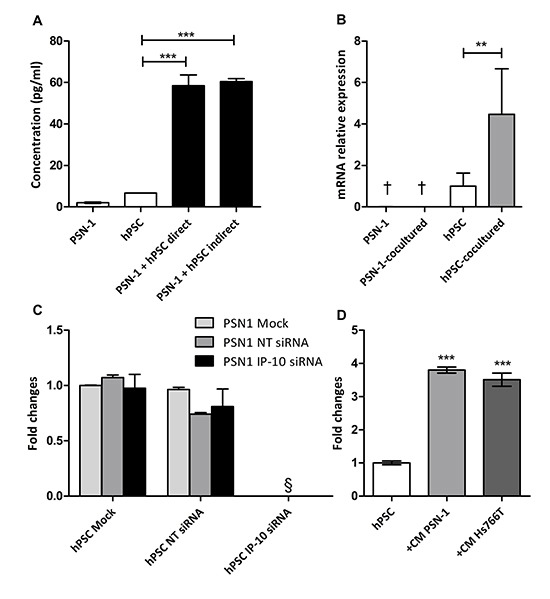
Induction of IP-10 expression does not require cell contact in coculture **(A)** IP-10 concentration in the supernatants from monocultures, direct and indirect cocultures was determined by ELISA. **(B)** IP-10 mRNA levels in monoculture and after coculture of PSN-1 and hPSC separated by a membrane (6 hr) assayed by qPCR. Values were normalized to GAPDH. **(C)** PSN-1 and hPSC were transfected with IP-10 siRNA and coculture conditions were set up as indicated 24 hr later. IP-10 expression in the coculture was evaluated with ELISA after 48 hr. NT, non targeting. **(D)** IP-10 concentration in the supernatant of hPSC with and without conditioned medium from PCCs determined by ELISA. The graph is representative of four independent experiments. Data are shown as mean ± SD. One-way ANOVA followed by Bonferroni's post-test: **, *p* < 0.001; ***, *p* < 0.0001. §, not detected. †, below limit of detection.

We asked which cell type was responsible for IP-10 production and whether the induction could be triggered by soluble factors. To ask about the nature of the trigger for induction, PCCs and PSCs were plated in the same well, but separated by a 0.1-μm pore permeable membrane, thus allowing the passage of soluble factors but not cells between compartments. This indirect coculture of PSN-1 with hPSC resulted in increased IP-10 levels to a similar extent as direct coculture (Figure 2A). Quantitative PCR analysis performed on PSN-1 and hPSC cells 6 hr after indirect coculture showed ~4-fold increased *IP-10* mRNA in cocultured hPSC while no increase was observed in cocultured PSN-1 cells (Figure 2B). In addition, siRNA knockdown of IP-10 in hPSC abrogated the increase in IP-10 protein levels, while siRNA knockdown in PSN-1 did not affect IP-10 levels in the cocultures (Figure 2C). These experiments demonstrate that PSCs are the major source of IP-10 in the cocultures and implicate soluble factors derived from the tumor cells in the induction. To extend this data, hPSC cells were cultured in conditioned media derived from PSN-1 and Hs766T monocultures. IP-10 secreted by hPSC cells increased by 3.8- and 3.5-fold, respectively after 48 hr (Figure 2D). Taken together, these data demonstrated that PCCs supply one or more soluble molecules that promote the expression and secretion of IP-10 by PSCs.

### IP-10 is not sufficient to promote proliferation and migration of PCCs

**Figure 3:**
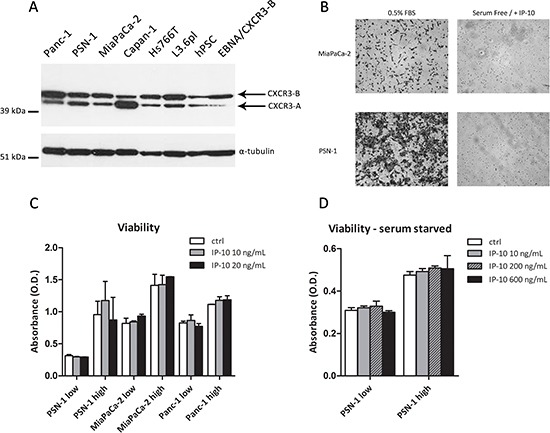
Functional effects of IP-10 on PCCs **(A)** Expression of CXCR3 in PCCs, PSCs and HEK293-EBNA/CXCR3-B analyzed by Western Blot. α-tubulin was used as loading control. **(B)** Microphotographs of PCCs migrated through the Boyden chamber in response to 0.5% FBS or IP-10 (24 hr). **(C-D)** Viability of PCCs after IP-10 addition determined using the MTT assay. Low/high refers to cell seeding density. Data are representative of two/three independent experiments. Data in the histograms are presented as mean ± SD.

CXCR3-binding ligands are known to stimulate proliferation and migration of tumor cells in glioma, colorectal carcinoma, prostate cancer, breast cancer and melanoma [14–19]. In order to investigate whether IP-10 could evoke a response in PCCs, we examined the protein expression of its cognate receptor CXCR3 in PCCs and PSCs. We detected CXCR3 expression on the surface of all MiaPaCa-2 cells and on a subset of Panc-1, PSN-1 and hPSC cells (Suppl. Figure 3). In addition, we analyzed its expression by western blot. A band around 45 kDa, corresponding to the molecular weight of CXCR3-B isoform, was present in Panc-1, PSN-1, MiaPaCa-2, Capan-1, L3.6pl, Hs766T and hPSC cells (Figure 3A) [20]. A band of approximately 40 kDa, corresponding to the smaller CXCR3-A isoform, was detected as well in all the cells (PCCs and PSCs) and was particularly abundant in Capan-1 (Figure 3A).

Because CXCR3 is present in a large percentage of PCCs, we investigated the activation of IP-10/CXCR3 signalling in PCCs. We analysed ERK and Akt phosphorylation, which are reported to be regulated by the activation of CXCR3 [21, 22]. Panc-1 and PSN-1 cells were stimulated with IP-10, but neither P-Akt nor P-ERK1/2 levels differed from unstimulated cells (Suppl. Figure 4A-B). Since the presence of serum could preclude IP-10 from activating CXCR3 signalling, cells were also serum-starved prior to stimulation. Capan-1, MiaPaCa-2, PSN-1 and Panc-1 cells were then exposed to IP-10 but levels of P-Akt and P-ERK1/2 showed no changes (Suppl. Figure 4C).

Despite not detecting a direct effect of IP-10 on CXCR3 signalling pathway, we decided to explore the possibility of a functional effect of IP-10 on pancreatic cancer cells. Therefore, we analyzed its chemotactic and proliferative effects on PCCs. In the chemotactic assay, IP-10 did not stimulate the migration of MiaPaCa-2 or PSN-1 cells (Figure 3B). Furthermore, IP-10 did not change proliferation of PCCs 96 h after stimulation, independent of seeding density and serum conditions (Figure 3C-D). Additionally, we tested the effects of IP-10 on migration and proliferation of PSCs. IP-10 (100-1000 ng/mL) did not promote hPSC migration in vitro, and viability of hPSC remained unchanged over 96 hr in the presence of IP-10 (data not shown). Taken together, these experiments showed that IP-10 had little or no direct effects on proliferation or migration of PCCs and PSCs *in vitro* but rather may have indirect functional effects in pancreatic cancer progression.

### IP-10 and CXCR3 expression are elevated in human PDAC

**Figure 4:**
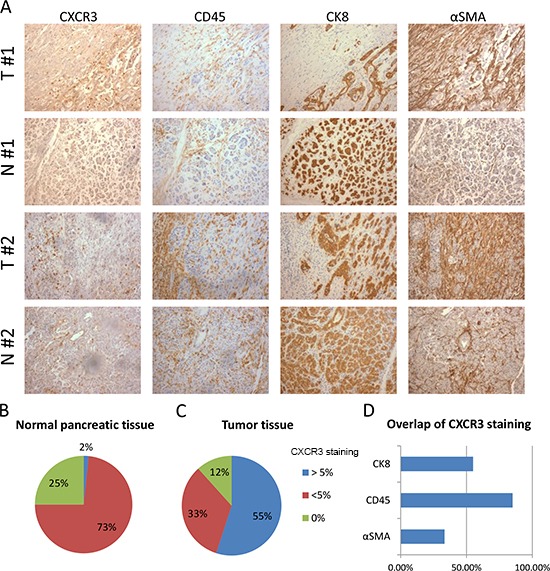
Expression of CXCR3 is elevated in PDAC compared to normal pancreas and is associated with leukocytes infiltration **(A)** The panel shows representative staining for CXCR3, α-SMA, CD45 and CK8 for two patients (patient #1, patient #2). For each patient one area of the tumor (T) tissue is compared with one of the normal (N) tissue surrounding the tumor. **(B-C)** Pie charts represent the percentages of normal pancreatic tissue (B) and tumor tissue (C) that have none, more or less than 5% area stained positive for CXCR3. **(D)** The graph represents the percentage of tumors where colocalization is seen between CXCR3 and CD45, CK8 or α-SMA. Only tumors where CXCR3 is expressed in more than 5% of total area were analyzed.

We analyzed the expression of CXCR3 by immunohistochemistry in pancreatic ductal adenocarcinoma samples of 30 patients. In addition, we compared the staining of CXCR3 and CD45 (pan-leukocyte marker), CK8 (pancreatic epithelial marker) and α-smooth muscle actin (α-SMA, marker of activated fibroblasts/PSCs) between the tumor and the adjacent normal tissue for each patient (Figure 4A). We found CXCR3 positivity in 5–35% of tumor area in 17 out of 30 patients, whilst 9 out of 30 patients had less than 5% CXCR3 positivity in tumor tissue and 4 patients showed no CXCR3 expression (Figure 4C). In the corresponding normal tissue, 22 out of 30 samples had very little expression of CXCR3 (less than 5% of total area were positive for CXCR3), and only one sample showed CXCR3 expression in a wider area whilst 7 samples had no CXCR3 expression (Figure 4B). The expression patterns of CXCR3 in tumors were quite different; some tumors (55%) expressed CXCR3 in CK8+ epithelial cancer cells, some (33%) in the stroma, but most tumors (85%) showed a scattered expression pattern of CXCR3 throughout the tissue, which overlapped with the expression of the pan-leukocyte marker CD45 (Figure 4A, D). Altogether, these data demonstrate increased expression of CXCR3 in pancreatic cancer and that CXCR3 is primarily expressed by tumor-infiltrating leukocytes.

**Figure 5:**
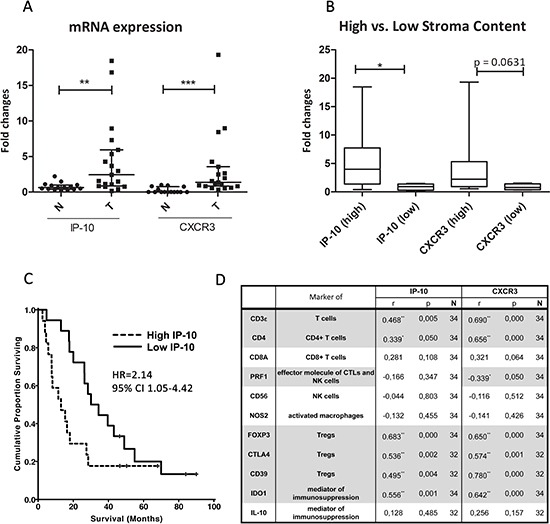
IP-10 and CXCR3 mRNA expression and correlation with leukocyte markers in PDAC **(A)** mRNA levels of IP-10 and CXCR3 in PDAC tumors (T; *n* = 19) compared to the adjacent normal pancreatic tissue (N; *n* = 15). Line indicates the median with the interquartile range. **(B)** Amount (high/low) of stroma content in PDAC directly associates with the expression of IP-10 and CXCR3. IP-10 and CXCR3 levels were measured by qPCR, normalized to endogenous GAPDH control and fold changes calculated over the expression levels in pancreatic samples from normal control tissue. Mann Whitney test *, *p* < 0.05; **, *p* < 0.001; ***, *p* < 0.0001. **(C)** Kaplan-Meier analyses of patients with resected PDAC stratified by high and low expression levels of IP-10 (*n* = 48). HR, Hazard ratio; CI, confidence interval. **(D)** Spearman's rho correlation between mRNA levels of the indicated genes, and IP-10 and CXCR3 levels in PDAC and normal pancreatic tissue. r, strength of the correlation; p, p-value; N, number of samples. Grey highlight indicates statistically significant correlation: *, *p* < 0.05; **, *p* < 0.001; ***, *p* < 0.0001; ns, not significant.

Furthermore, we determined *IP-10* and *CXCR3* mRNA expression levels in 19 PDAC and 15 normal (adjacent to tumor tissues) pancreatic tissue samples by qPCR. Median mRNA expression of *IP-10* and *CXCR3* was greater in PDAC patient samples than normal tissue by 3.8- and 77-fold on average respectively (Figure 5A). Moreover, levels of *IP-10* correlated with *CXCR3* expression (Spearman's rho: *r* = 0.625, *p* < 0.0001) suggesting an association between the presence of the ligand and its cognate receptor. Histopathological analysis of the tumors showed that PDAC containing high stromal desmoplasia expressed higher *IP-10* mRNA levels (*p* = 0.029) and tended to have a higher expression of *CXCR3* when compared to normal tissue (*p* = 0.063) (Figure 5B). Overall, we observed increased levels of mRNA expression for *IP-10* and *CXCR3* in PDAC compared to normal pancreatic tissue which is associated with increased amount of stroma present in the tumors.

### High IP-10 expression levels correlate with decreased survival in PDAC patients

To explore the clinical relevance of IP-10, we examined the expression levels of IP-10 in an independent cohort of 48 patients diagnosed with PDAC, in which mRNA microarray analysis of tumor tissues was performed and published by Dozynkiewicz and colleagues [23]. Clinical characteristics of patients are described in Supplementary Table 3. In this dataset, *IP-10* expression was 2.18-fold greater in the PDAC specimens compared to normal tissue (*p* < 0.04, t-test). Moreover, using the median *IP-10* mRNA expression value of the PDAC specimens as a cutoff, the cohort stratified into two groups with high expression levels of IP-10 in the tumor correlated with significantly shorter survival (*p* = 0.045, Log-rank test) (Figure 5C). The median overall survival of patients with low IP-10 expression was 30.1 months (95% CI 17.5–42.7) compared to 12.0 months (95% CI 3.2-16.8) for patients with high IP-10 levels. In order to elucidate the relation between IP-10 expression and stroma in human pancreatic cancer, *IP-10* expression was normalized to the expression levels of α-SMA, a marker for activated fibroblasts and PSCs. Patients were then stratified again in two groups using the median *IP-10/α-SMA* value. The group with high *IP-10/α-SMA* ratio had significantly shorter overall survival (p = 0.015, Log-rank test) than those with low *IP-10/α-SMA* ratio (Suppl. Figure 5) indicating that IP-10 expression is not simply reflecting the percentage of stroma but it is an independent prognostic marker. The finding that IP-10 upregulation correlated with shorter survival provides evidence that IP-10 plays a functional role in pancreatic cancer. In addition, these data suggest that IP-10 levels may be used as a biomarker to predict survival in patients with resectable pancreatic ductal adenocarcinoma.

### IP-10 and CXCR3 expression in PDAC correlates with the presence of tregs

IP-10 is a known chemotactic factor for leukocytes, in particular, T cells. In order to test a potential association between IP-10 and leukocyte infiltration into tumors, we evaluated the mRNA expression levels of markers expressed by different leukocyte subpopulations in 19 PDAC and 15 normal (adjacent to the tumor) pancreatic tissue samples by qPCR. These included CD3ε, CD4, CD8A, perforin 1 (pore forming protein 1, PRF1), neural cell adhesion molecule 1 (NCAM1, also called CD56), nitric oxide synthase 2 (NOS2 or iNOS), FOXP3, CTLA4, CD39 (also called ENTPD1), indoleamine 2,3-dioxygenase 1 (IDO1) and IL-10.

*CD3ε*, *CD8A*, *CD56* and *NOS2* mRNA levels were similar in PDAC and normal tissue (Suppl. Figure 6A, C, I-J). However, median *CD4* levels increased by approximately 1.7-fold in PDAC compared to controls while *PRF1* was decreased nearly 2-fold (Suppl. Figure 6B, D). This suggests that there are more CD4^+^ T cells in the pancreatic tumor compared to normal tissue. The decrease in the levels of *PRF1*, a key effector molecule for cytolysis mediated by T and NK cells, in presence of *CD8A* and *CD56* expression in PDAC suggests that the cytotoxic activity or the number of CD8^+^ T and NK cells may be reduced in the tumor. Median levels of *FOXP3*, *CTLA4* and *CD39*, used here as surrogate markers for Tregs, were increased by approximately 16, 15 and 3-fold respectively, in PDAC tumors compared to normal tissue (Suppl. Figure 6E-G). Also, mRNA levels of *IDO1* were increased by about 13-fold in tumors compared to normal controls, while no significant changes were observed for *IL-10* expression levels (Suppl. Figure 6H, K). Subsequently, we evaluated the association between expression of these markers and levels of *IP-10* and *CXCR3* in all samples. We found that *IP-10* and *CXCR3* levels correlated with the expression of *CD3ε*, *CD4*, *FOXP3*, *CTLA4*, *CD39* and *IDO1*, molecules characteristically expressed by Tregs (Figure 5D). *CXCR3* expression also correlated with a decrease in *PRF1* levels (Figure 5D). Overall, the transcriptional analysis for leukocyte markers in PDAC samples indicates an association between IP-10 expression and its receptor CXCR3 with the presence of Treg and an immune-suppressed PDAC microenvironment.

### IP-10 stimulates chemotaxis of CD4^+^ and CD8^+^ T cells as well as Tregs isolated from the circulation of patients with PDAC

**Figure 6:**
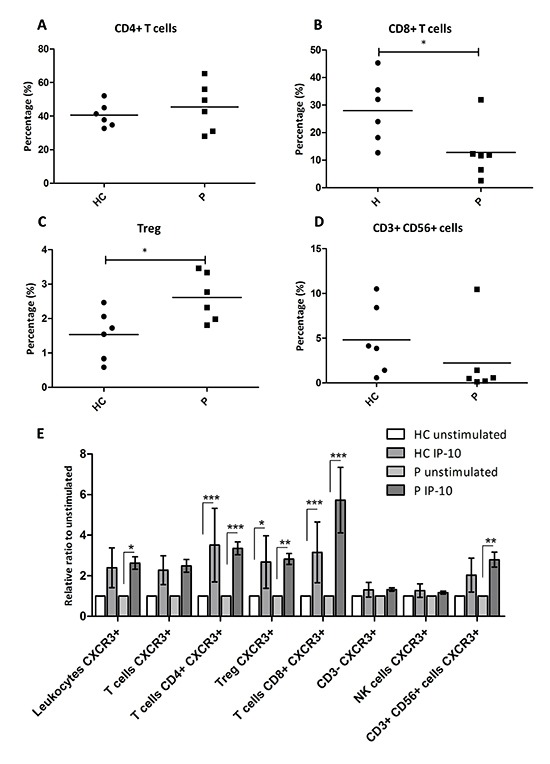
IP-10 recruits CXCR3^+^ effector T cells as well as CXCR3^+^ Tregs **(A-D)** Percentage of peripheral leukocytes that are CD4^+^ T cells (A), CD8^+^ T cells (B), CD3^+^ CD56^+^ cells (D) and the percentage of T cells that are Tregs (C) among healthy controls and patients with PDAC. Mann-Whitney U test: *, *p* < 0.05. **(E)** Relative ratio of migrating leukocytes in response to IP-10 over the number in the unstimulated controls. Two-way ANOVA followed by Bonferroni's post test: *, *p* < 0.05; **, *p* < 0.001; ***, *p* < 0.0001. HC, Healthy control; P, patients with PDAC.

To test whether IP-10 induced chemotaxis of circulating leukocytes, we collected PBMCs from healthy volunteers (*n* = 6) and patients with PDAC (*n* = 6) and performed an *ex vivo* chemotactic assay. After incubation with and without IP-10, all migrated cells were collected and stained. The flow cytometry parameters and CXCR3 staining used to distinguish the different leukocyte subsets are shown in Supplementary Figure 7A-B. Notably, we identified subsets of CD4^+^ T cells and CD8^+^ T cells expressing CXCR3 receptor on the cell surface and also subsets of Tregs (CD3^+^CD4^+^CD25^+^FoxP3^+^) and of CD3^+^ CD56^+^ cells expressing CXCR3 (Suppl. Figure 7B). As previously reported, the percentage of circulating Tregs was increased in PDAC patients compared to healthy volunteers (Figure 6C) [24]. In contrast, the percentage of circulating CD8^+^ T cells was lower in patients with PDAC while the overall percentage of CD4^+^ T cells and CD3^+^ CD56^+^ cells was similar between the two groups (Figure 6A-B, D).

In the presence of IP-10, PBMCs from PDAC patients showed a statistically significant increase in the numbers of migrating CXCR3^+^ leukocytes (Figure 6E, *p* < 0.05). IP-10 stimulated the recruitment of CXCR3^+^ CD4^+^ and CD8^+^ T cells as well as CXCR3^+^ Tregs in the PBMCs from PDAC patients but not from healthy controls (Figure 6E). Interestingly, the numbers of CD3^+^ CD56^+^ CXCR3^+^ cells were also augmented significantly by IP-10, but only in leukocytes from PDAC patients (Figure 6E). Taken together, these data demonstrate that IP-10 can act as a chemoattractant for CXCR3^+^ Tregs suggesting that they share IP-10 chemotactic mechanisms with other effector T cells. The higher number of circulating CXCR3^+^ Tregs in PDAC patients may result from stromal induction of IP-10 and may also result in a higher number of infiltrating Tregs in the tumor. Furthermore, IP-10 was shown to recruit CD3^+^ CD56^+^ cells expressing CXCR3.

## DISCUSSION

Since PSCs are the most prominent cell type in the extensive stromal reaction typical of PDAC, we asked whether we could identify specific factors involved in the interaction between PSCs and PCCs. As an initial approach, we compared the expression of a panel of cytokines and growth factors in the supernatant of PSCs compared to conventional fibroblasts in order to characterize the PSC-specific profile. Together with other factors already known to be secreted by PSCs such as IL-8, IL-6 and SDF-1α, we found that human PSCs secrete LIF, IL-1Rα, IL-15, GROα, eotaxin and IP-10. Subsequently, by comparing the expression of cytokines and growth factors from monoculture and coculture supernatants of PSCs and PCCs, we gained further insights into tumor-stroma interactions establishing that PCCs regulate IP-10 expression by PSCs.

IP-10 was significantly upregulated after coculture of PSCs with five of seven different PCCs. PSCs were the main source of IP-10 in the coculture as its transcription was upregulated specifically in PSCs but not in PCCs, and its secretion was abrogated following knock down of IP-10 in PSCs. IP-10, also called CXCL10, is a chemokine implicated in diverse processes such as inflammation and angiogenesis and it is expressed in patients with a wide range of diseases [25]. IP-10 is a known chemoattractant for activated T cells. The effects of IP-10 are mediated through binding to the CXCR3 receptor. The isoform A of CXCR3 is the receptor for two other IFN-γ inducible chemokines: MIG/CXCL9 and I-TAC/CXCL11 [26]. IP-10 together with platelet factor-4/CXCL4 also binds with high affinity to the isoform B of CXCR3 [20]. High levels of IP-10 and CXCR3 have been associated with chronic pancreatitis but a role for IP-10 in pancreatic cancer has not been previously reported [27].

Accumulating data implicate CXCR3 signaling in promotion of tumor growth, migration and invasion. For example, inhibition of CXCR3 signaling by either gene silencing or small molecule inhibitors decreased the number of pulmonary metastases formed by breast cancer cells [17, 28]. Activation of CXCR3 signaling was reported to promote tumor growth of glioma and basal cell carcinoma cell lines and to enhance invasion of melanoma and colorectal carcinoma cells [15, 18, 19, 29]. Using flow cytometry and western blotting analysis, we detected CXCR3 expression on the plasma membrane of PCCs and both CXCR3-A and –B isoforms were expressed at equivalent levels. However, PCCs did not migrate or proliferate in response to IP-10 stimulation in our experimental setting. Furthermore, IP-10 stimulation did not induce ERK or Akt activation in PCCs, suggesting that IP-10 may have little direct effects on PCCs. A possible explanation for these different biological outcomes between cancer cell types may rely on the fact that PCCs express equivalent levels of CXCR3-A and CXCR3-B isoforms. Indeed, activation of Ras was shown to downregulate CXCR3-B expression in breast cancer cell lines, which led to upregulation of IP-10 and increased proliferation, likely through activation of the remaining CXCR3-A isoform [16]. In prostate cancer cell lines, the ability of CXCR3-binding chemokines to induce migration was also dependent on aberrant expression of CXCR3-A and downregulation of CXCR3-B [14]. Since most PCCs express CXCR3-B at similar levels as CXCR3-A, this may counteract overall CXCR3 signaling. Another plausible explanation for our findings is that other chemokines that bind CXCR3, present in pancreatic cancer microenvironment, may mediate migration, invasion and growth of PCCs instead of IP-10. It may also be the case that in PCCs, CXCR3 is uncoupled from intracellular signalling and thus IP-10 does not modify the PCCs cellular response. Furthermore, a recent study reported the truncation of 2 amino acids at the N-terminal of IP-10 generated by the dipeptidyl peptidase IV (DPP4 or CD26) [30]. The resulting cleaved form of IP-10 is a dominant negative form of the protein, which is capable of binding CXCR3 but does not induce signalling [30]. We detected the expression of DPP4 in a number of PCCs (data not shown), hence this may be another possible explanation of why PCCs were not responsive to IP-10 despite the expression of the cognate receptor.

*IP-10* and *CXCR3* mRNA levels were upregulated in pancreatic adenocarcinoma of patients obtained from pancreatectomy compared to normal pancreatic tissue, in two independent groups of patients. Furthermore, we found that *IP-10* and *CXCR3* levels in human PDAC correlated with higher tumor desmoplasia, suggesting that IP-10 expression may depend on the presence of PSCs in the tumor microenvironment similar to our *in vitro* model. Importantly, patients with surgically resected PDAC who displayed high intratumoral expression of *IP-10* had shorter median overall survival by 18.1 months compared to patients with lower *IP-10* levels.

These data raise questions about the potential mechanisms through which IP-10 may be affecting patient outcome. In our gene expression analysis of PDAC samples, we found that levels of both *IP-10* and its receptor *CXCR3* correlated with markers of Tregs (*i.e. CD4, FOXP3, CTLA4* and *CD39*) within PDAC tissue but did not correlate with markers of CD8^+^ T cells. We also detected high expression of *IDO1*, a potent immunosuppressive molecule, in association with markers of Tregs and high levels of *IP-10* and *CXCR3* within PDAC. Tregs can suppress the activity of CD4^+^ Th1 and effector cytotoxic T lymphocytes (CTLs) in the peripheral tissue where antigen is presented [31, 32]. Tregs can also induce dendritic cells (DCs) to produce IDO1 through the interaction of CTLA4 with CD80 and CD86 molecules expressed by DCs [33]. The correlation between IP-10 and markers of Tregs, together with the local expression of *IDO1* suggest the presence of an immunosuppressive environment within the PDAC tissues where IP-10 is upregulated. In addition, we found that *CXCR3* expression levels were inversely correlated with *PRF1*, an effector molecule of CTLs and NK cells. Thus, it is likely that *IP-10* and *CXCR3* expression may be associated with the presence of functionally active Tregs, which in turn, may contribute to immunosuppression by blocking the cytotoxic activity of CTLs or NK.

Furthermore, we established that IP-10 is a chemoattractant for CXCR3^+^ CD4^+^ T cells and CD8^+^ T cells as well as CXCR3^+^ Tregs isolated from blood samples of PDAC patients and healthy volunteers. Interestingly, we also found that IP-10 was capable of recruiting a subset of T cells expressing CD3, CD56 and CXCR3 exclusively in PBMC derived from PDAC patients. These cells are NKT-like cells whose role in tumors remains to be elucidated. Several studies have shown that *IP-10* expression correlates with activity of activated T cells or other leukocyte subsets against different tumor types [34–37]. For example, in melanoma samples, IP-10 expression was associated with the number of CXCR3^+^ lymphocytes and correlated with spontaneous regression of melanoma lesions [34]. In colorectal cancer, high levels of IP-10 protein led to increased recruitment of effector CD8^+^ T cells and predicted longer recurrence-free survival [35]. IP-10 was found in 45% of human breast cancers and was associated with CXCR3 expression and lymphocytic infiltration [38]. Thus, the presence of IP-10 in solid tumors is often considered a good prognostic factor due to their ability to recruit activated lymphocytes. On the other hand, a recent study reported that Tregs expressing CXCR3 and the transcriptional factor typical of Th1 immunity T-bet accumulated in response to Th1-type inflammatory stimuli. This in turn blocked Th1 immune response in human ovarian cancer *ex vivo* [39, 40]. It was proposed that CXCR3^+^ Tregs may respond to CXCR3-ligands to control strong Th1 and CTLs activation, in order to prevent an autoimmune response. This hypothesis is supported by the fact that in a mouse model of autoimmune hepatitis, adoptive transfer of CXCR3^+^ Tregs can induce tolerance and ameliorate the disease [41]. Likewise, other subsets of Tregs expressing Th lineage-specific molecules were shown to suppress the activity of the correspondent Th subset [42,43]. In agreement with previous studies, we found that the presence of Tregs was increased in PDAC compared to normal pancreatic tissue [24, 44, 45]. Furthermore, the balance between the ratio of peripheral Tregs and total T cells is altered in pancreatic cancer patients [24]. Our data show that PDAC patients have on average double the number of Tregs in the peripheral blood compared to healthy controls. Thus, increased expression of IP-10 in pancreatic cancer tissue, although would lead to recruitment of CXCR3^+^ Tregs and CXCR3^+^ CD4^+^ and CD8^+^ T cells as shown in our *in vitro* assay, may preferentially contribute to increased infiltration of Tregs in the tumor due to the elevated numbers of circulating Tregs, hence inhibiting tumor immunoresponse.

It is important to consider that in other tumor types such as breast cancer and melanoma, IP-10 may drive an anti-tumoral response by recruiting CXCR3^+^ Th1 lymphocytes [34, 35]. These opposing effects could be attributed to an altered balance between CXCR3^+^ Th1 and CXCR3^+^ Tregs. The imbalance between Tregs and CD8^+^ T cells in the circulation of pancreatic cancer patients could explain the results found in different cancer types. We have to consider also that CXCR3 is a promiscuous receptor and that, in addition to IP-10, it can bind CXCL11/I-TAC, CXCL9/Mig and CXCL4/PF-4 [46]. The presence of these chemokines in the tumour microenvironment could have additional effects on immune cell recruitment. Moreover, the way different chemokines affect the immune response may vary between tumor entities and even between patients and further experiments are needed to understand the role of this chemokine network in PDAC.

Based on our findings, we propose a model in which stromal expression of IP-10, induced by PCCs, recruits immunosuppressive CXCR3^+^ Tregs into PDAC. Tregs may then block the stimulation of T effector cells by antigen presenting cells, and/or directly inhibit T effector and NK cells, consequently favoring pancreatic tumor growth and progression. In conclusion, this study suggests that IP-10 upregulation in pancreatic cancer stroma may regulate recruitment of lymphocytes and consequently the immunoresponse against the tumor in a context-dependent manner. Additionally, it indicates the potential use of IP-10 as a biomarker to predict survival in patients with resectable pancreatic adenocarcinoma. Validation of this model can provide novel insights regarding the physiological and pathological functions of IP-10, and can have important implication for the development of new multimodal therapeutic approaches.

## MATERIALS AND METHODS

### Cell culture

Human Panc-1, MiaPaCa-2, Capan-1, Capan-2, Hs766T and MRC5 were purchased from American Type Culture Collection, PSN-1 was obtained through an MTA with Merck & Co. and L3.6pl was kindly provided by Prof. I. Fidler (University of Texas, USA). These cell lines were grown in Dulbecco's Modified Eagle's Medium (DMEM; Sigma-Aldrich) with 10% FBS and penicillin/streptomycin (P/S). The human pancreatic stellate cells (hPSC) were obtained through a MTA with Prof. A. Masamune (Tohoku University, Japan) and cultured in Ham's F12/DMEM 1:1 with 10% FBS and P/S. HEK293-EBNA/CXCR3-B cells were kindly provided by Prof. L. Lasagni (University of Florence, Italy) and cultured in DMEM 10% FBS, P/S supplemented with G418 and Hygromycin B from Invitrogen-Life Technologies. All cells were routinely tested for mycoplasma contamination using the MycoAlert^®^ Assay kit (Lonza).

### Multiplex cytokines analysis

Monocultures of 4×10^4^ Panc-1, 4×10^5^ hPSC and 4×10^5^ MRC5 cells and coculture of Panc-1 with hPSC (or MRC5) were established by plating the cells alone or together in 5 mL media. The conditioned media was collected 24 hr after culture and the concentration of 50 cytokines, growth factors and chemokines in conditioned media of PSCs and PCCs was assessed with the Human group I 27-Plex and the Human group II 23-Plex cytokines arrays (Bio-Rad Laboratories). Data were acquired using a Luminex 100 plate reader and analyzed with the associated Bio-Plex^®^ Manager 5.0 software. The concentration of each factor in each sample was inferred from the exponential phase of the standard curve determined for each cytokine.

### ELISA

The concentrations of IP-10 and GROα in culture supernatants were measured by ELISA using the human IP-10 and Duoset GROα ELISA Sets (#550926 BD Bioscience and #DY275 R&D Systems, respectively) according to manufacturer's instructions.

### RNA isolation

RNA isolation from adherent cells was performed using TRIzol (Life Technologies) according to manufacturer's instructions, followed by DNAse treatment (Life Technologies).

Human tissue samples were obtained with approval of the local ethics committee and with informed, written consent from the patients. Thirty samples were obtained from patients with pancreatic carcinoma admitted to Surgical Department (Technische Universitaet Muenchen, Germany). These included 18 PDAC tissues (median age 68 years, range 42–83) and 12 normal peri-tumoral pancreatic tissues, of which 10 paired with PDAC samples. Median follow-up after surgery was 16.9 months. Histology-guided sample selection was performed by a pathologist to identify tumor and to exclude cancer cell contamination in the control samples. The classification of high and low stroma content was determined by P.K.J. Additionally, three normal peri-tumoral pancreatic tissues and one PDAC tissue were collected from patients admitted to the Churchill Hospital (Oxford University Hospitals NHS Trust, UK). Tissues were homogenized and RNA was isolated using the RNeasy Kit (Qiagen).

### qPCR

cDNA preparation was performed according to standard procedures, using the RT-PCR ProtoScript M-Mulv kit (New England Biolabs). qPCR was performed using the TaqMan Gene Expression Master Mix (Life Technologies). Predesigned and pre-optimized TaqMan Gene Expression Assays (Life Technologies) used are listed in Suppl. Table 1. Relative measurement of the amplified product was performed using the comparative threshold cycles method by normalizing to endogenous GAPDH control and then calculating the fold-induction over the expression levels of a control sample (calibrator).

### siRNA transfection

siGENOME non targeting pool #2 and human IP-10 ON-TARGETplus SMART pool were purchased from Dharmacon (Thermo Scientific) and siRNA transfections were performed following the manufacturer's instructions. Briefly, cells were seeded in 6-well plate in complete media overnight followed by the addition of siRNA mixed with INTERFERin (Polyplus Transfection). The cells were further incubated 24 hr before being plated for coculture experiments.

### Immunoblot analysis

Lysates were assayed by Western blot analysis for the expression of CXCR3 and α-tubulin using the antibodies #MAB160 (R&D Systems) and #14-4502 (eBioscience), respectively. Akt antibody #9272, P-Akt (Ser 473) antibody #9271, ERK1/2 antibody #90101, P-ERK1/2 (phospho-p44/42) antibody #90102 were purchased from Cell Signaling. GAPDH antibody #G9545 was obtained from Sigma-Aldrich.

### Cell growth assay

Cell growth was assessed with CellTiter 96 AQ_ueous_ Non-Radioactive assay (Promega) accordingly to manufacturer's recommendations. Cells were seeded in 96-well plates with or without IP-10 (10-600 ng/mL; PeproTech) in triplicates for each condition. After 4 days of culture, MTS solution was added and absorbance measured after 3 hr.

### Peripheral blood mononuclear cells isolation

PBMCs were isolated from the blood of healthy donors and patients with PDAC using Lymphoprep™ density gradient medium in Sepmate™-50 tubes (StemCell Technologies). Isolated PBMCs were immediately frozen in FBS with 10% DMSO until further use. Samples were obtained under the approval of the Oxford Radcliffe Biobank research tissue bank ethics and after informed, written consent of the patients.

### Migration assays

Migration studies with PCCs were performed using cell culture inserts for 24-well plate with 8-μm pores (BD Biosciences). Cells were cultured overnight in 0.5% FBS medium then detached by gentle mechanical scraping. Cells were resuspended in serum free DMEM and placed in the inserts for 24 hr in the absence or presence of IP-10 (0.25-600 ng/mL) in the bottom chamber. Experiments were done in triplicates. Cells that adhered to the membrane were fixed and stained with Hematoxylin and crystal violet.

Migration of PBMCs was performed using inserts for 24-well plate with 5-μm pores (Corning Life Sciences). Human PBMCs from patients with pancreatic cancer were thawed overnight in 2μl/mL Benzonase (Novagen) in RPMI with 10% heat inactivated FBS. PBMCs were not activated in order to preserve their biological cell activity as close as possible to the patients' peripheral tissue. The following day, cells were starved for 20 min in RPMI with 0.1% FBS and 0.1% BSA. Afterwards, PBMCs were counted, resuspended in RPMI 0.1% FBS and 3 × 10^5^ cells in 100 μL were added to the top of the Boyden Chamber. The bottom chamber contained 700 μL RPMI (1% FBS and 0.1% BSA) with or without rIP-10 (200 ng/mL). All conditions were done in triplicates. Transwells were then incubated for 3 hr and cells that had transmigrated into the lower chamber were collected, stained with antibody for flow cytometry analysis and resuspended in a solution of PBS/0.2% BSA containing an equal amount of Calibrite Beads (BD Bioscience), used for normalization. Leukocyte subpopulations were characterized by flow cytometry and the count of each leukocyte subtype was normalized to the number of Calibrite Beads in each sample.

### Immunohistochemistry

Paraffin-embedded tissue microarray (TMA) of pancreatic cancer tissues versus corresponding normal tissues (AccuMax Array; Stretton Scientific) was deparaffinised, immersed in 10 mM citric buffer (pH 6.0) and microwaved for 10 min. The slide was washed and endogenous peroxidase activity was quenched by incubating the slide in 0.3% hydrogen peroxide for 20 min followed by successive blocking with TNB (Perkin Elmer) for 30 min. The slide was incubated overnight at 4°C with primary antibodies diluted in TNB. After 3 washes with PBS, the slide was incubated with biotinylated secondary antibody. Peroxidase staining (DAB substrate kit for peroxidase) was performed as recommended by the manufacturer (Vector Laboratories, Inc). The primary antibodies employed were against CXCR3 #C1366 (Sigma), α-SMA #M0851 (Dako), cytokeratin 8 (CK8) #AM142-5M (Biogenex) and CD45 #ab8216 (Abcam). Staining was scored independently by SL and SYY to determine CXCR3 positivity and overlap of CXCR3 staining with α-SMA, CK8 and CD45.

### Flow cytometry

Antibodies used to detect cell surface markers of PBMCs were: anti-human CD3-Alexa Fluor 700, anti-human CD4-FITC, anti-human CD25-APC-eFluor 780, anti-human CD39-PE-Cy7 and anti-human CD56-PE from eBioscience; anti-human CD8-Pacific Blue, anti-human CXCR3 PE-Cy5 and PE-Cy5 mouse IgG1, κ isotype control from BD Bioscience.

Cells were resuspended in 5 μl Live/Dead solution (Invitrogen-Life Technologies) for 10 min at RT, then incubated with cell surface antibodies for 30 min at RT followed by intracellular staining for Foxp3 using the anti-mouse/rat/human FOXP3 (Biolegend) following the manufacturer's instructions. Flow cytometry analysis was performed using a LSRII (BD Bioscience) and data analyzed with FlowJo software.

## SUPPLEMENTARY FIGURES AND TABLES



## Abbreviations

α-SMA: α-smooth muscle actin
CK8: cytokeratin 8
CTLs: cytotoxic T lymphocytes
DCs: dendritic cells
ECM: extracellular matrix
GRO-α: melanoma growth stimulating activity-α
IDO1: indoleamine 2,3-dioxygenase 1
IP-10: interferon-γ inducible protein 10
LIF: leukemia inhibitory factor
MDSCs: myeloid-derived suppressor cells
NCAM1: neural cell adhesion molecule 1
NOS2: nitric oxide synthase 2
PBMCs: peripheral blood mononuclear cells
PCCs: pancreatic cancer cells
PDAC: pancreatic ductal adenocarcinoma
SDF-1α: stromal cell-derived factor-1α
PRF1: perforin 1
PSCs: pancreatic stellate cells
TMA: tissue microarray
Tregs: regulatory T cells

## References

[1] Siegel R, Naishadham D, Jemal A (2013). Cancer statistics, 2013. CA Cancer J Clin.

[2] Ferlay J, Parkin DM, Steliarova-Foucher E (2010). Estimates of cancer incidence and mortality in Europe in 2008. Eur J Cancer.

[3] Vonlaufen A, Joshi S, Qu C, Phillips PA, Xu Z, Parker NR, Toi CS, Pirola RC, Wilson JS, Goldstein D, Apte MV (2008). Pancreatic stellate cells: partners in crime with pancreatic cancer cells. Cancer Res.

[4] Provenzano PP, Cuevas C, Chang AE, Goel VK, Von Hoff DD, Hingorani SR (2012). Enzymatic targeting of the stroma ablates physical barriers to treatment of pancreatic ductal adenocarcinoma. Cancer Cell.

[5] Mantoni TS, Lunardi S, Al-Assar O, Masamune A, Brunner TB (2011). Pancreatic stellate cells radioprotect pancreatic cancer cells through beta1-integrin signaling. Cancer Res.

[6] Clark CE, Hingorani SR, Mick R, Combs C, Tuveson DA, Vonderheide RH (2007). Dynamics of the immune reaction to pancreatic cancer from inception to invasion. Cancer Res.

[7] Tang D, Yuan Z, Xue X, Lu Z, Zhang Y, Wang H, Chen M, An Y, Wei J, Zhu Y, Miao Y, Jiang K (2012). High expression of Galectin-1 in pancreatic stellate cells plays a role in the development and maintenance of an immunosuppressive microenvironment in pancreatic cancer. Int J Cancer.

[8] Mace TA, Ameen Z, Collins A, Wojcik S, Mair M, Young GS, Fuchs JR, Eubank TD, Frankel WL, Bekaii-Saab T, Bloomston M, Lesinski GB (2013). Pancreatic Cancer-Associated Stellate Cells Promote Differentiation of Myeloid-Derived Suppressor Cells in a STAT3-Dependent Manner. Cancer Res.

[9] Ene-Obong A, Clear AJ, Watt J, Wang J, Fatah R, Riches JC, Marshall JF, Chin-Aleong J, Chelala C, Gribben JG, Ramsay AG, Kocher HM (2013). Activated pancreatic stellate cells sequester CD8+ T cells to reduce their infiltration of the juxtatumoral compartment of pancreatic ductal adenocarcinoma. Gastroenterology.

[10] Kraman M, Bambrough PJ, Arnold JN, Roberts EW, Magiera L, Jones JO, Gopinathan A, Tuveson DA, Fearon DT (2010). Suppression of antitumor immunity by stromal cells expressing fibroblast activation protein-alpha. Science.

[11] Ozdemir BC, Pentcheva-Hoang T, Carstens JL, Zheng X, Wu CC, Simpson TR, Laklai H, Sugimoto H, Kahlert C, Novitskiy SV, De Jesus-Acosta A, Sharma P, Heidari P (2014). Depletion of carcinoma-associated fibroblasts and fibrosis induces immunosuppression and accelerates pancreas cancer with reduced survival. Cancer Cell.

[12] Masamune A, Kikuta K, Watanabe T, Satoh K, Hirota M, Hamada S, Shimosegawa T (2009). Fibrinogen induces cytokine and collagen production in pancreatic stellate cells. Gut.

[13] Gao Z, Wang X, Wu K, Zhao Y, Hu G (2010). Pancreatic stellate cells increase the invasion of human pancreatic cancer cells through the stromal cell-derived factor-1/CXCR4 axis. Pancreatology.

[14] Wu Q, Dhir R, Wells A (2012). Altered CXCR3 isoform expression regulates prostate cancer cell migration and invasion. Mol Cancer.

[15] Liu C, Luo D, Reynolds BA, Meher G, Katritzky AR, Lu B, Gerard CJ, Bhadha CP, Harrison JK (2011). Chemokine receptor CXCR3 promotes growth of glioma. Carcinogenesis.

[16] Datta D, Flaxenburg JA, Laxmanan S, Geehan C, Grimm M, Waaga-Gasser AM, Briscoe DM, Pal S (2006). Ras-induced modulation of CXCL10 and its receptor splice variant CXCR3-B in MDA-MB-435 and MCF-7 cells: relevance for the development of human breast cancer. Cancer Res.

[17] Ma X, Norsworthy K, Kundu N, Rodgers WH, Gimotty PA, Goloubeva O, Lipsky M, Li Y, Holt D, Fulton A (2009). CXCR3 expression is associated with poor survival in breast cancer and promotes metastasis in a murine model. Mol Cancer Ther.

[18] Zipin-Roitman A, Meshel T, Sagi-Assif O, Shalmon B, Avivi C, Pfeffer RM, Witz IP, Ben-Baruch A (2007). CXCL10 promotes invasion-related properties in human colorectal carcinoma cells. Cancer Res.

[19] Kawada K, Sonoshita M, Sakashita H, Takabayashi A, Yamaoka Y, Manabe T, Inaba K, Minato N, Oshima M, Taketo MM (2004). Pivotal role of CXCR3 in melanoma cell metastasis to lymph nodes. Cancer Res.

[20] Lasagni L, Francalanci M, Annunziato F, Lazzeri E, Giannini S, Cosmi L, Sagrinati C, Mazzinghi B, Orlando C, Maggi E, Marra F, Romagnani S, Serio M (2003). An alternatively spliced variant of CXCR3 mediates the inhibition of endothelial cell growth induced by IP-10, Mig, and I-TAC, and acts as functional receptor for platelet factor 4. J Exp Med.

[21] Aksoy MO, Yang Y, Ji R, Reddy PJ, Shahabuddin S, Litvin J, Rogers TJ, Kelsen SG (2006). CXCR3 surface expression in human airway epithelial cells: cell cycle dependence and effect on cell proliferation. Am J Physiol Lung Cell Mol Physiol.

[22] Ji R, Lee CM, Gonzales LW, Yang Y, Aksoy MO, Wang P, Brailoiu E, Dun N, Hurford MT, Kelsen SG (2008). Human type II pneumocyte chemotactic responses to CXCR3 activation are mediated by splice variant A. Am J Physiol Lung Cell Mol Physiol.

[23] Dozynkiewicz MA, Jamieson NB, Macpherson I, Grindlay J, van den Berghe PV, von Thun A, Morton JP, Gourley C, Timpson P, Nixon C, McKay CJ, Carter R, Strachan D (2012). Rab25 and CLIC3 collaborate to promote integrin recycling from late endosomes/lysosomes and drive cancer progression. Dev Cell.

[24] Vizio B, Novarino A, Giacobino A, Cristiano C, Prati A, Ciuffreda L, Montrucchio G, Bellone G (2012). Potential plasticity of T regulatory cells in pancreatic carcinoma in relation to disease progression and outcome. Exp Ther Med.

[25] Groom JR, Luster AD (2011). CXCR3 ligands: redundant, collaborative and antagonistic functions. Immunol Cell Biol.

[26] Salcedo R, Resau JH, Halverson D, Hudson EA, Dambach M, Powell D, Wasserman K, Oppenheim JJ (2000). Differential expression and responsiveness of chemokine receptors (CXCR1-3) by human microvascular endothelial cells and umbilical vein endothelial cells. FASEB J.

[27] Singh L, Bakshi DK, Majumdar S, Vasishta RK, Arora SK, Wig JD (2007). Expression of interferon-gamma- inducible protein-10 and its receptor CXCR3 in chronic pancreatitis. Pancreatology.

[28] Walser TC, Rifat S, Ma X, Kundu N, Ward C, Goloubeva O, Johnson MG, Medina JC, Collins TL, Fulton AM (2006). Antagonism of CXCR3 inhibits lung metastasis in a murine model of metastatic breast cancer. Cancer Res.

[29] Lo BK, Yu M, Zloty D, Cowan B, Shapiro J, McElwee KJ (2010). CXCR3/ligands are significantly involved in the tumorigenesis of basal cell carcinomas. Am J Pathol.

[30] Casrouge A, Decalf J, Ahloulay M, Lababidi C, Mansour H, Vallet-Pichard A, Mallet V, Mottez E, Mapes J, Fontanet A, Pol S, Albert ML (2011). Evidence for an antagonist form of the chemokine CXCL10 in patients chronically infected with HCV. J Clin Invest.

[31] Tang Q, Adams JY, Tooley AJ, Bi M, Fife BT, Serra P, Santamaria P, Locksley RM, Krummel MF, Bluestone JA (2006). Visualizing regulatory T cell control of autoimmune responses in nonobese diabetic mice. Nat Immunol.

[32] McLachlan JB, Catron DM, Moon JJ, Jenkins MK (2009). Dendritic cell antigen presentation drives simultaneous cytokine production by effector and regulatory T cells in inflamed skin. Immunity.

[33] Munn DH, Sharma MD, Mellor AL (2004). Ligation of B7-1/B7-2 by human CD4+ T cells triggers indoleamine 2,3-dioxygenase activity in dendritic cells. J Immunol.

[34] Wenzel J, Bekisch B, Uerlich M, Haller O, Bieber T, Tuting T (2005). Type I interferon-associated recruitment of cytotoxic lymphocytes: a common mechanism in regressive melanocytic lesions. Am J Clin Pathol.

[35] Muthuswamy R, Berk E, Junecko BF, Zeh HJ, Zureikat AH, Normolle D, Luong TM, Reinhart TA, Bartlett DL, Kalinski P (2012). NF-kappaB hyperactivation in tumor tissues allows tumor-selective reprogramming of the chemokine microenvironment to enhance the recruitment of cytolytic T effector cells. Cancer Res.

[36] Sun H, Kundu N, Dorsey R, Jackson MJ, Fulton AM (2001). Expression of the Chemokines IP-10 and Mig in IL-10 Transduced Tumors. J Immunother (1991).

[37] Giese NA, Raykov Z, DeMartino L, Vecchi A, Sozzani S, Dinsart C, Cornelis JJ, Rommelaere J (2002). Suppression of metastatic hemangiosarcoma by a parvovirus MVMp vector transducing the IP-10 chemokine into immunocompetent mice. Cancer Gene Ther.

[38] Mulligan AM, Raitman I, Feeley L, Pinnaduwage D, Nguyen LT, O'Malley FP, Ohashi PS, Andrulis IL (2013). Tumoral lymphocytic infiltration and expression of the chemokine CXCL10 in breast cancers from the Ontario Familial Breast Cancer Registry. Clin Cancer Res.

[39] Koch MA, Tucker-Heard G, Perdue NR, Killebrew JR, Urdahl KB, Campbell DJ (2009). The transcription factor T-bet controls regulatory T cell homeostasis and function during type 1 inflammation. Nat Immunol.

[40] Redjimi N, Raffin C, Raimbaud I, Pignon P, Matsuzaki J, Odunsi K, Valmori D, Ayyoub M (2012). CXCR3+ T Regulatory Cells Selectively Accumulate in Human Ovarian Carcinomas to Limit Type I Immunity. Cancer Res.

[41] Lapierre P, Beland K, Yang R, Alvarez F (2012). Adoptive transfer of ex vivo expanded regulatory T cells in an autoimmune hepatitis mouse model restores peripheral tolerance. Hepatology.

[42] Yamazaki T, Yang XO, Chung Y, Fukunaga A, Nurieva R, Pappu B, Martin-Orozco N, Kang HS, Ma L, Panopoulos AD, Craig S, Watowich SS, Jetten AM (2008). CCR6 regulates the migration of inflammatory and regulatory T cells. J Immunol.

[43] Zheng Y, Chaudhry A, Kas A, deRoos P, Kim JM, Chu TT, Corcoran L, Treuting P, Klein U, Rudensky AY (2009). Regulatory T-cell suppressor program co-opts transcription factor IRF4 to control T(H)2 responses. Nature.

[44] Liyanage UK, Moore TT, Joo HG, Tanaka Y, Herrmann V, Doherty G, Drebin JA, Strasberg SM, Eberlein TJ, Goedegebuure PS, Linehan DC (2002). Prevalence of regulatory T cells is increased in peripheral blood and tumor microenvironment of patients with pancreas or breast adenocarcinoma. J Immunol.

[45] Hiraoka N, Onozato K, Kosuge T, Hirohashi S (2006). Prevalence of FOXP3+ regulatory T cells increases during the progression of pancreatic ductal adenocarcinoma and its premalignant lesions. Clin Cancer Res.

[46] Singh UP, Venkataraman C, Singh R, Lillard JW (2007). CXCR3 axis: role in inflammatory bowel disease and its therapeutic implication. Endocr Metab Immune Disord Drug Targets.

