# Supplementary material for: IP-10/CXCL10 induction in human pancreatic cancer stroma influences lymphocytes recruitment and correlates with poor survival
Source: Oncotarget. 2014 Nov 25;5(22):11064–80. doi: 10.18632/oncotarget.2519 (PMC4294325; doi:10.18632/oncotarget.2519)
Supplement: Supplementary file 1 [file oncotarget-05-11064-s001.pdf]

SUPPLEMENTARY FIGURES AND TABLES

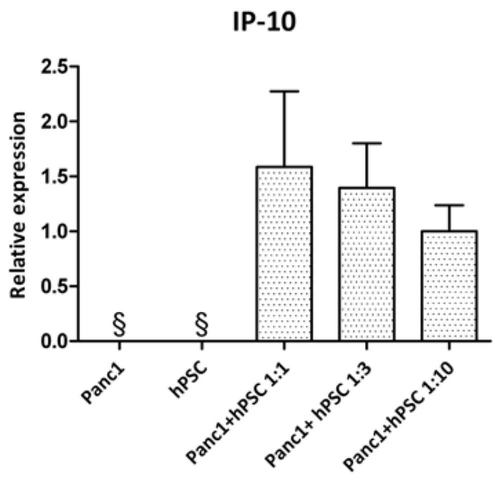

Supplementary Figure S1: IP-10 secretion by PSCs cocultured with cancer cells compared to monoculture. Levels of IP-10 in Panc-1 and hPSC monoculture or coculture at the indicated Panc-1:hPSC ratio cocultured for 24 hr was analyzed by ELISA. Relative expression of concentration normalized to Panc-1+hPSC 1:10 ratio (*n*=3). §, not detected.

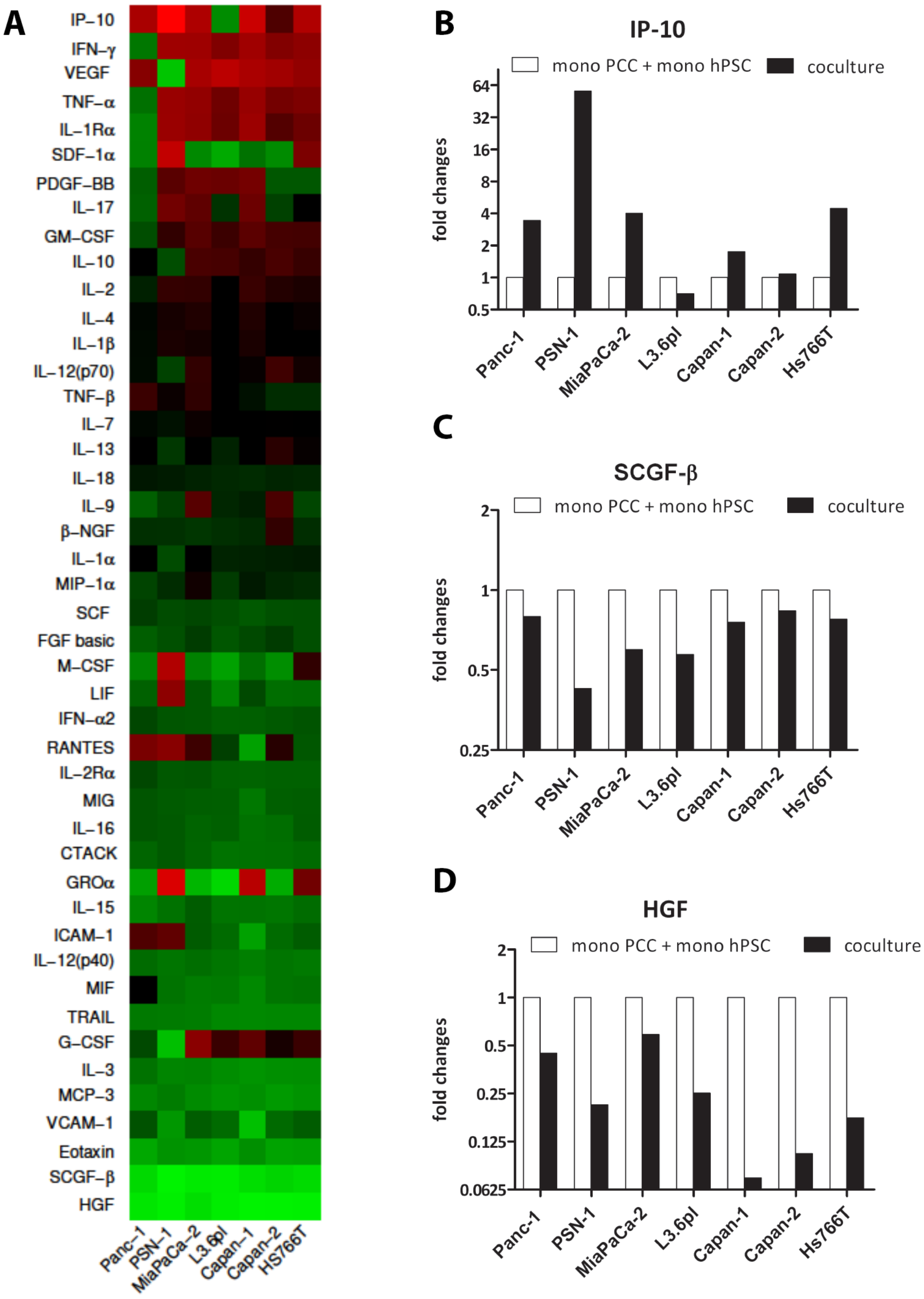

Supplementary Figure S2: Comparison of different PCC lines cocultured with PSCs. Expression of cytokine and growth factors was measured using the Human group I 27-Plex cytokines array and the Human group II 23-Plex cytokines array in the cell-free supernatant of PCCs (25×10^3) cultured with and without hPSC (25×10^3) for 48 hr in 24-well plate (500 µL volume). **(A)** Concentration of factors in monoculture (mono PCCs + mono hPSC) was subtracted from coculture for each factor. Values are represented in a heat map with increased intensity color code where red is more than zero and green is below zero (*n*=1). **(B-D)** Relative expression of each coculture normalized to the sum of the respective monocultures for IP-10, SCGF-β and HGF (*n*=1).

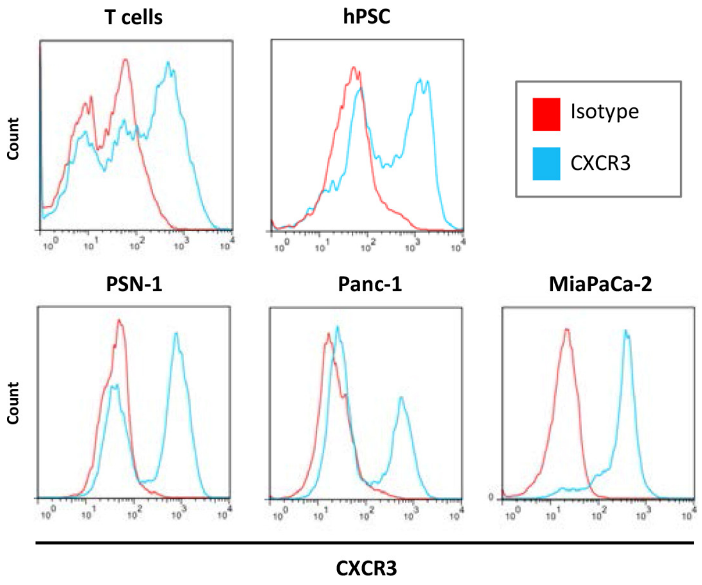

Supplementary Figure S3: Surface Expression of CXCR3 in PCCs and hPSC. FACS analysis of CXCR3 surface expression. PCCs and hPSC were cultured in complete media. T cells were used as positive control. Cells were grown until 80% confluent in 6-wells plate and then detached by gentle mechanical scraping. Cells were washed once with PBS and resuspended 1×10^6 cells/mL. For each test 100 µL of the cell suspension was blocked for 15 min at RT using human IgG (Sigma-Aldrich) at a concentration of 1 mg/mL in PBS 2% FBS. Cells were stained for 20 min at RT by using a directly conjugated anti-human CXCR3-APC antibody (5 µL/test; #561732, BD Bioscience) and mouse IgG1, κ-APC as isotype control (5 µl/test; #555751, BD Biosciences). After one wash with PBS 2% FBS the cells were resuspended and analyzed using FACS Calibur (BD Biosciences) and data elaborated with FlowJo software (Tree Star). Data are representative of three independent experiments.

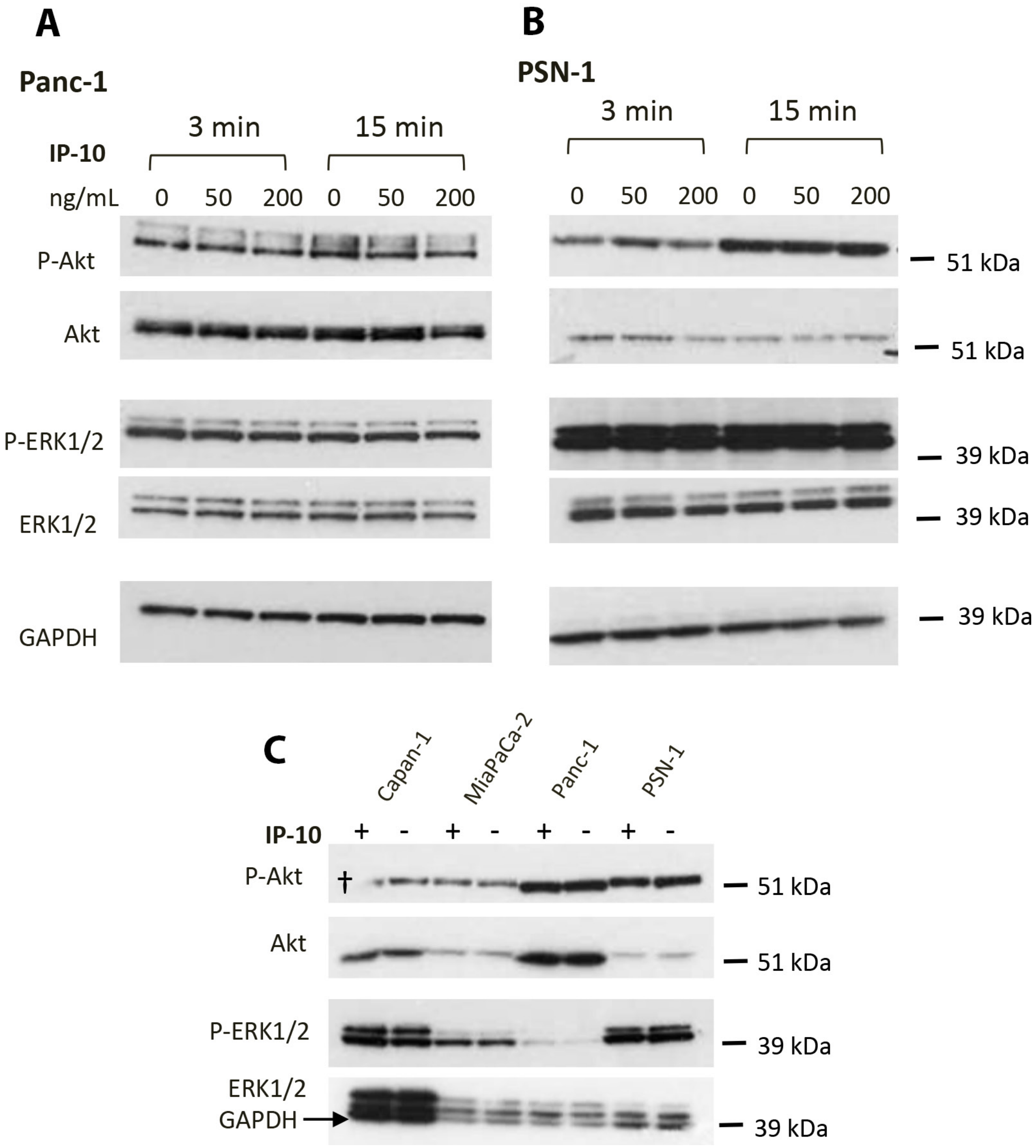

Supplementary Figure S4: Analysis of P-Akt and P-ERK1/2 levels upon IP-10 stimulation. Western blot of P-Akt, P-ERK1/2, total Akt and ERK. GAPDH was used as loading control. **(A-B)** Panc-1 (A) and PSN-1 (B) were stimulated with IP-10 using the concentrations and time indicated. **(C)** PCC were starved over night in 0.1% FBS media and then exposed to IP-10 (600 ng/ml) for 20 min. †, unspecific missing signal due to membrane damage.

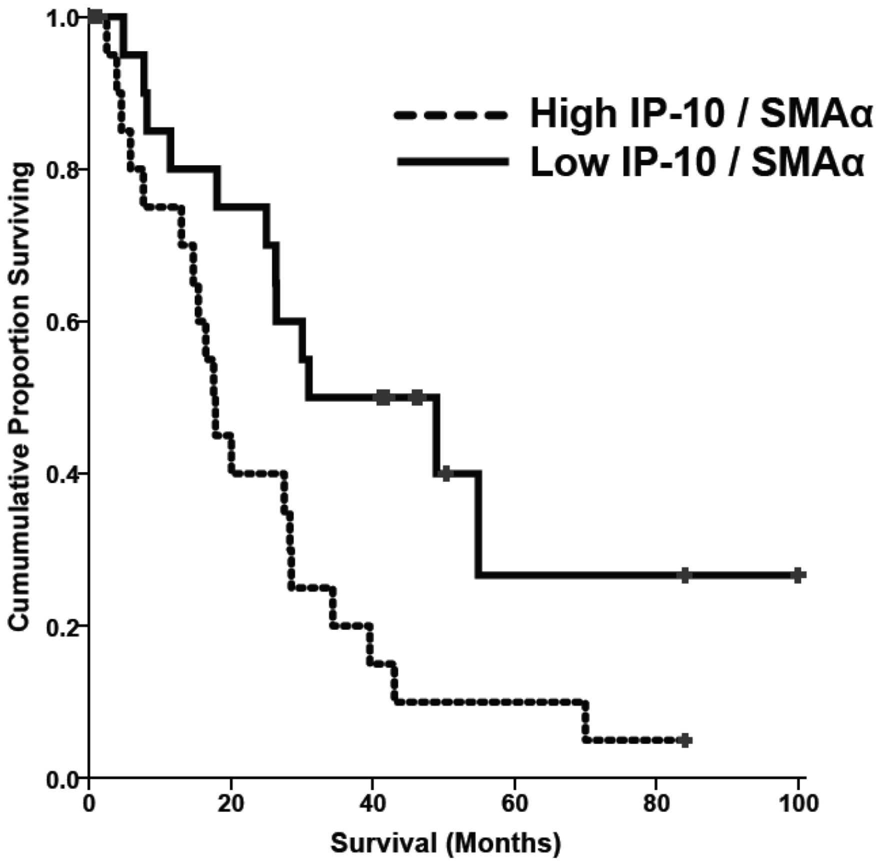

Supplementary Figure S5: Overall survival of patients with pancreatic cancer based on IP-10/α-SMA gene expression. Kaplan-Meier analyses of patients with resected PDAC stratified by high and low ratio of IP-10/α-SMA (*n*=48).

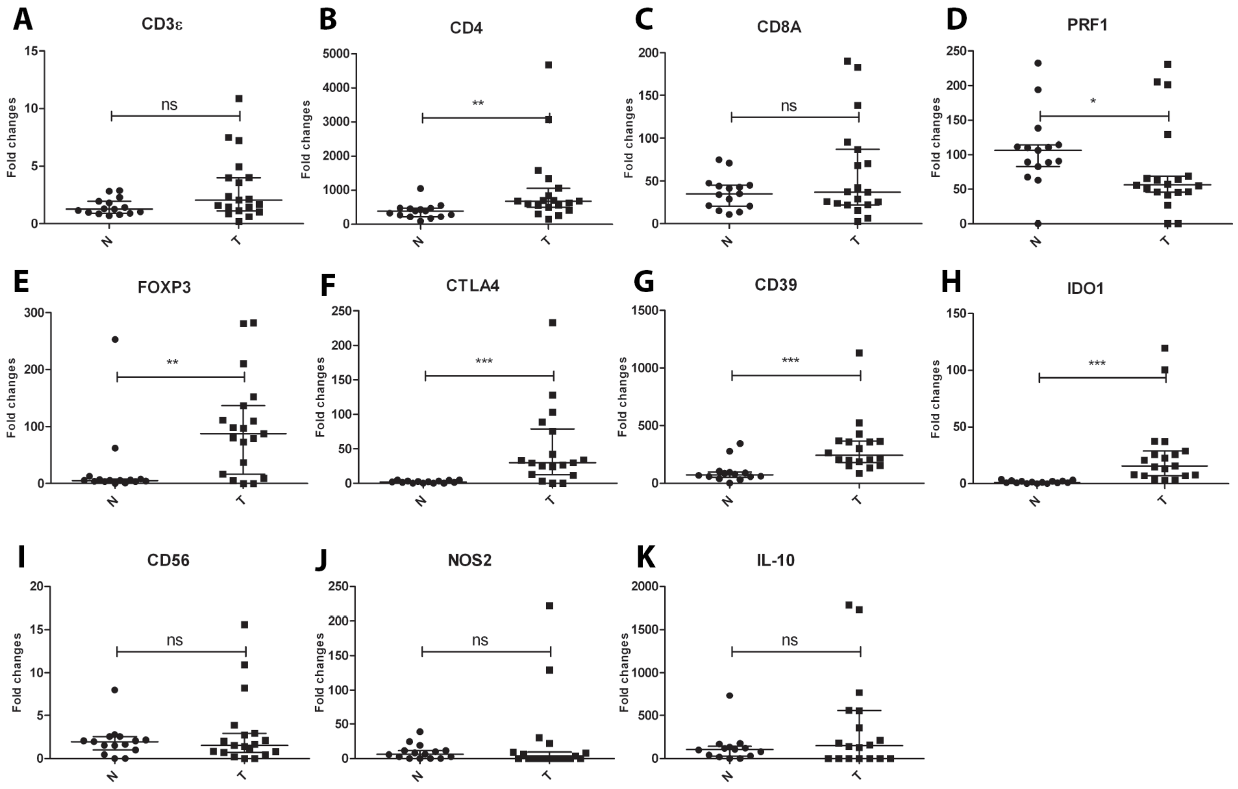

Supplementary Figure S6: mRNA expression levels of markers for leukocyte subpopulation in PDAC. (A-K) mRNA levels of CD3ε, CD4, CD8A, PRF1, CD56, NOS2, FOXP3, CTLA4, CD39, IL-10 and IDO1 in PDAC tumors (T; *n*=19) compared to the para-normal pancreatic tissue (N; *n*=15). Line indicates the median with the interquartile range. Markers were measured by qPCR, normalized to the endogenous GAPDH control and fold changes calculated over the expression levels in a Human Reference Total RNA library. In F, G and K the number of samples analyzed for this factors are 14 for N and 18 for T. Mann Whitney test *, $p<0.05$; **, $p<0.001$; ns, non-significant.

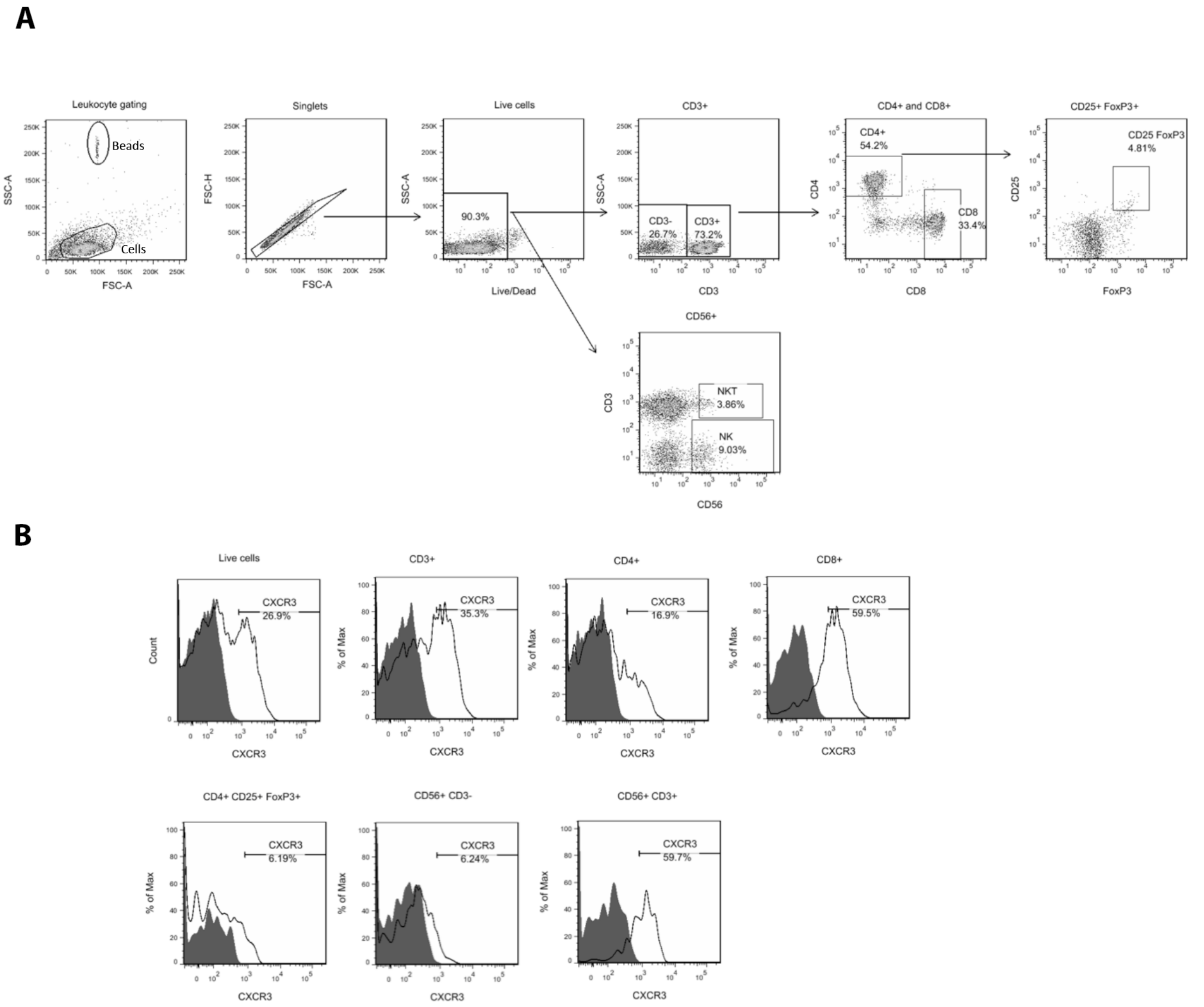

Supplementary Figure S7: Flow cytometry analysis of PBMC. (A) Gating strategy used to determine the different leukocytes subpopulation. **(B)** FACS analysis of CXCR3 surface expression in the different subpopulation.

Supplementary Table S1. List of TaqMan assays

Gene	TaqMan assay ID
Human CXCL10 (IP-10)	Hs00171042_m1, FAM™ Dye
Human CXCR3	Hs01847760_s1, FAM™ Dye
Human GAPDH	Hs99999905_m1, FAM™ Dye
Human GAPDH	GAPDH Endogenous Control, VIC™ Dye
Human CD3E (CD3ε)	Hs01062241_m1, FAM™ Dye
Human CD4	Hs01058407_m1, FAM™ Dye
Human CD8A	Hs00233520_m1, FAM™ Dye
Human NCAM1 (CD56)	Hs00941830_m1, FAM™ Dye
Human PRF1	Hs00169473_m1, FAM™ Dye
Human NOS2	Hs01075529_m1, FAM™ Dye
Human IDO1	Hs00984148_m1, FAM™ Dye
Human FOXP3	Hs01085834_m1, FAM™ Dye
Human CTLA4	Hs03044418_m1, FAM™ Dye
Human ENTPD1 (CD39)	Hs00969559_m1, FAM™ Dye
Human IL-10	Hs00961622_m1, FAM™ Dye

Supplementary Table S2. Concentration of cytokines and growth factors determined with the Human group I 27-Plex cytokines array and the Human group II 23-Plex cytokines array. Data are expressed as concentration of proteins (pg/mL) ± SD after 24 hr of culture (*n*=4). Factors highlighted in bold were significantly increased in monoculture of hPSC compared to MRC5. ND, not detected.

Analyte	Panc-1 Mean ± SD (pg/mL)			Panc-1 + hPSC Mean ± SD (pg/mL)			hPSC Mean ± SD (pg/mL)			Panc-1 + MRC5 Mean ± SD (pg/mL)			MRC5 Mean ± SD (pg/mL)		
**Interleukins**															
**IL-8**	**46.0**	±	**19.2**	**28,101.0**	±	**9,354.2**	**28,101.0**	±	**9,354.2**	**484.4**	±	**108.2**	**463.9**	±	**157.6**
**IL-6**	**ND**			**> 31,094.0**			**> 31,094.0**			**1,001.7**	±	**753.0**	**993.0**	±	**769.8**
**LIF**	**15.3**	±	**2.7**	**338.1**	±	**42.4**	**344.3**	±	**65.3**	**43.0**	±	**16.7**	**41.6**	±	**12.8**
**IL-1Rα**	**ND**			**223.1**	±	**42.2**	**225.3**	±	**37.6**	**39.3**	±	**15.6**	**36.0**	±	**13.1**
**IL-15**	**12.0**	±	**6.2**	**67.4**	±	**8.2**	**67.7**	±	**4.5**	**26.8**	±	**5.1**	**13.9**	±	**0.5**
IL-2	ND			16.9	±	2.2	16.9	±	3.0	3.9	±	2.9	3.6	±	2.9
IL-17	9.9	±	1.4	63.7	±	16.7	66.1	±	17.9	14.9	±	7.2	15.8	±	7.0
IL-4	ND			11.3	±	1.2	10.7	±	1.3	2.7	±	1.9	2.7	±	1.8
IL-2Rα	1.5	±	1.1	94.3	±	10.0	97.5	±	5.0	29.3	±	17.6	33.5	±	18.1
IL-16	ND			121.5	±	10.3	133.0	±	4.1	53.2	±	39.3	59.9	±	38.3
IL-9	10.1	±	2.0	43.3	±	6.3	43.3	±	6.9	20.7	±	5.0	19.7	±	5.6
IL-12(p40)	11.2	±	2.2	163.1	±	19.4	178.8	±	13.6	73.6	±	36.8	81.9	±	43.7
IL-1a	0.7	±	0.3	6.0	±	1.6	6.0	±	1.7	2.4	±	2.1	2.8	±	2.1
IL-3	27.4	±	2.9	219.6	±	20.2	228.8	±	7.3	121.1	±	76.7	116.5	±	73.5
IL-7	ND			12.7	±	5.1	11.9	±	5.0	6.4	±	2.9	6.5	±	3.6
IL-18	ND			15.1	±	2.3	16.4	±	2.1	9.2	±	4.0	9.6	±	3.5
IL-12(p70)	0.7	±	0.4	14.1	±	3.1	14.1	±	4.1	10.9	±	0.9	10.8	±	1.1
IL-10	1.2	±	0.9	35.3	±	10.7	35.4	±	9.6	28.6	±	2.7	27.4	±	2.2
IL-13	ND			14.8	±	5.7	16.1	±	8.0	13.3	±	3.9	14.5	±	5.9
IL-1β	ND			6.8	±	0.4	6.9	±	0.7	ND			ND		
IL-5	ND			ND			ND			ND			ND		
**Chemokines/monokines**															
**GROα**	**127.4**	±	**57.5**	**13,754.3**	±	**7,216.0**	**13,358.4**	±	**7,719.6**	**177.8**	±	**69.4**	**129.2**	±	**58.3**
**Eotaxin**	**57.1**	±	**14.4**	**5,999.2**	±	**4,106.9**	**6,037.3**	±	**4,086.3**	**136.0**	±	**29.4**	**130.6**	±	**45.6**
**SDF-1α**	**49.6**	±	**30.3**	**3,208.7**	±	**1,368.8**	**3,056.3**	±	**752.4**	**126.4**	±	**37.2**	**80.6**	±	**25.6**
**IP-10**	**43.7**	±	**32.9**	**16,472.9**	±	**2,379.3**	**2,930.1**	±	**1,608.6**	**2,374.6**	±	**1,161.5**	**337.0**	±	**76.8**
MCP-3	ND			1,020.5	±	468.9	988.8	±	415.8	149.4	±	73.2	165.0	±	82.0
MIG	ND			30.6	±	5.5	34.1	±	2.8	18.2	±	9.3	21.2	±	11.2
RANTES	25.5	±	5.7	295.9	±	20.8	158.9	±	3.9	71.3	±	17.1	46.8	±	23.5
MIP-1α	5.8	±	0.2	23.6	±	1.1	23.1	±	1.3	10.2	±	2.1	10.0	±	2.8
MIF	37.0	±	15.3	409.8	±	43.9	426.4	±	50.7	244.8	±	128.5	245.0	±	122.8
CTACK	ND			246.4	±	62.6	259.3	±	67.9	170.7	±	103.1	170.0	±	100.3
MCP-1	>2,288.8			>4,842.5			>6,413.8			>4,781.3			>1,725.1		
MIP-1β	19.5	±	8.2	29.6	±	2.0	27.3	±	9.5	ND			18.4	±	0.4

(*Continued*)

**Inflammatory cytokines**															
IFN-γ	14.6	±	3.4	2,462.7	±	312.0	2,390.0	±	243.4	342.5	±	260.6	345.8	±	274.9
TNF-α	2.0	±	0.8	354.2	±	32.6	340.9	±	27.0	73.4	±	48.6	73.6	±	54.0
TNF-β	1.9	±	0.2	8.1	±	3.1	8.4	±	3.2	5.1	±	5.5	4.9	±	5.2
IFN-α2		ND		62.7	±	4.6	65.9	±	4.4	39.0	±	17.4	40.9	±	17.1
**Growth factors**															
M-CSF	65.6	±	72.4	3,100.8	±	2,898.5	3,431.2	±	3,287.6	250.7	±	256.9	234.3	±	243.0
G-CSF	1.2	±	1.0	319.2	±	88.3	311.8	±	86.9	45.6	±	36.3	43.4	±	35.8
PDGF-BB	3.4	±	2.6	97.2	±	5.0	95.6	±	5.0	15.8	±	5.4	14.2	±	8.6
GM-CSF		ND		127.8	±	17.6	128.2	±	11.6	24.7	±	18.0	23.9	±	16.3
HGF	6.3	±	5.0	9,127.0	±	1,559.5	11,295.2	±	1,905.3	4,225.2	±	2,927.7	5,151.8	±	3,620.5
FGF basic	18.3	±	1.0	44.9	±	12.4	44.0	±	4.5	20.5	±	4.3	20.4	±	3.2
β-NGF		ND		24.8	±	9.9	27.1	±	10.5	14.0	±	3.9	14.8	±	3.9
SCF		ND		54.4	±	4.1	57.7	±	4.2	37.5	±	7.7	38.3	±	7.9
VEGF	29.6	±	18.0	2,036.3	±	570.7	1,881.8	±	885.4	1,406.0	±	161.8	1,355.0	±	170.7
SCGF-β	98.0	±	63.6	29,372.6	±	7,969.0	28,767.0	±	9,709.6	26,426.8	±	19,274.0	27,879.3	±	20,505.3
**Apoptosis Mediator**															
TRAIL	6.4	±	0.0	225.2	±	21.8	248.4	±	12.7	83.3	±	75.3	91.8	±	75.4
**Adhesion molecules**															
ICAM-1	41.5	±	30.7	164.0	±	67.2	120.7	±	28.7	76.9	±	43.6	35.3	±	12.4
VCAM-1	0.6	±	0.0	87.0	±	3.3	93.2	±	7.0	45.5	±	20.5	48.6	±	21.2

Supplementary Table S3. Clinical characteristics of PDAC patients in which the gene expression analysis was performed

Characteristic	48 patient cohort
Demographic	
Gender (F: M)	19/ 29
Age (≤ 65/ > 65 yrs)	25/ 23
Pathological	
Tumor stage (T2 / T3)	4/ 44
Lymph node metastasis (no / yes)	8/ 40
Tumor size (≤ 30 / > 30 mm)	28/ 20
Tumor grade (low / high)	32/ 16
Perineural invasion (no / yes)	3/ 45
Venous invasion (no / yes)	15/ 33
Lymphatic invasion (no / yes)	31/ 17
Operative, treatment and outcome	
Vascular resection (no / yes)	36/ 12
Resection margin status (R0 / R1)	10/ 38
Adjuvant therapy (no / yes)	20/ 28
Survival (months) (median / mean)	18.0/24.9
